# In RRP, serologic response to HPV is frequently absent and slow to develop

**DOI:** 10.1371/journal.pone.0230106

**Published:** 2020-03-11

**Authors:** Farrel J. Buchinsky, Nicole Ruszkay, William Valentino, Craig S. Derkay, John E. McClay, Robert W. Bastian, Charles M. Myer, Kevin W. Lollar, Dalya Guris

**Affiliations:** 1 Respiratory Papillomatosis Program, Allegheny Health Network, Pittsburgh, Pennsylvania, United States of America; 2 Drexel University College of Medicine, Philadelphia, Pennsylvania, United States of America; 3 Department of Otolaryngology Head Neck Surgery, Eastern Virginia Medical School, Norfolk, Virginia, United States of America; 4 Frisco ENT for Children, Dallas, Texas, United States of America; 5 Bastian Voice Institute, Downers Grove, Illinois, United States of America; 6 Department of Otolaryngology–Head and Neck Surgery, Cincinnati Children's Hospital Medical Center, Cincinnati, Ohio, United States of America; 7 Ear, Nose, & Throat Center of the Ozarks, Springdale, Arkansas, United States of America; 8 Merck & Co., Inc., (MSD), North Wales, Pennsylvania, United States of America; Albert Einstein College of Medicine, UNITED STATES

## Abstract

**Background:**

Recurrent respiratory papillomatosis (RRP) is characterized by repeated formation of papillomas in the respiratory tract and is caused by human papillomavirus (HPV) types 6 and 11. Women with genital HPV infection are slow to develop weak humoral immunity, but respond robustly to the HPV vaccine. We wondered if people with RRP had a similar immune response.

**Methods:**

A convenience cross-sectional sample of patients with RRP were recruited into one of four groups: 1) adults and adolescents with active RRP, 2) children with active RRP, 3) RRP patients who had undergone HPV vaccination prior to enrollment and, 4) people with RRP who were in remission. Anti-HPV6 and HPV11 serology was determined by cLIA on a single blood draw.

**Results:**

Of the 70 subjects enrolled, 36, 16, 8, and 10, were in groups 1, 2, 3, and 4, respectively. 47% of participants aged >11 years and 81% aged ≤11 years possessed no antibodies against HPV6 or HPV11 (ie. double seronegative). 61% of patients in remission were double seronegative. All participants who had received HPV vaccine previously were seropositive to at least one of these low risk HPV types (ie none of them were double seronegative). Among patients who had active RRP and never had HPV vaccination (n = 52) there was an association between duration of symptoms and seropositivity. Of those who were seropositive, the geometric mean duration of symptoms was 11 years compared to 4.7 years for those who were seronegative (p = 0.001).

**Conclusion:**

People with RRP are capable of developing a humoral response to HPV6 and HPV11. That response appears to be robust when initiated by the HPV vaccine, but either nonexistent or slow to develop in response to infection. Most in remission do not have demonstrable antibody levels against HPV6 or HPV11.

## Introduction

Recurrent respiratory papillomatosis (RRP) is a chronic disease that affects the airways of children and adults. The causal agent is low-risk human papillomavirus (HPV) types 6 and 11 and very rarely high-risk HPV types. The disease most often presents as papillomas on the vocal folds. Disease presentation ranges from mild hoarseness to severe airway obstruction and often recurs [1]. Standard treatment of RRP involves surgical removal of the papillomas via direct laryngoscopy and subsequent ablation. After several years of active disease and repeated surgeries, the course of the disease slows and most patients enter remission [2,3]. The most prominent theory for the development of RRP is peripartum inoculation upon contact with genital HPV infection [4]. The intrinsic and environmental factors that influence susceptibility to developing RRP and the clinical course of the disease remains to be determined.

HPV vaccines contain virus-like particles (VLP) made up entirely of type-specific outer coat L1 proteins. The vaccines induce potent humoral responses that are highly effective at preventing HPV infection of naive hosts. In 2006, the newly-approved quadrivalent HPV vaccine containing HPV6, 11, 16, and 18 VLPs (Gardasil^®^) prompted us to inquire how patients with RRP might respond to the vaccine and whether the serological titers would be comparable to those already infected with HPV. Thus, in 2007, the collaborators of this project set out to establish if patients with RRP possessed antibodies against HPV6 or 11 and to quantify the serological response. Furthermore, we wanted to determine if people with RRP were capable of the same robust response to the vaccine that had been seen of subjects in the pivotal efficacy trials. Several years have passed since the study was executed and the preliminary results were communicated via poster and oral presentation. Since then there has been interest in the feasibility of using the quadrivalent HPV (qHPV) vaccine as a treatment modality for RRP [5–10].

The current study is a descriptive study with no formal hypotheses. No intervention was conducted. The study thus does not address the therapeutic efficacy or preventative capacity of the qHPV vaccine for RRP. The purpose of this study was to assess HPV6 and 11 titers in patients diagnosed with RRP and to determine whether the qHPV vaccine elicited a robust serologic response similar to the one seen in people without RRP.

## Materials and methods

Five surgical practices recruited and enrolled participants: Eastern Virginia Medical School, Norfolk, VA; University of Texas—Southwestern, Dallas, TX; Bastian Voice Institute, Downer’s Grove, IL; University of Cincinnati, Cincinnati, OH; University of Missouri Hospital & Clinics, Columbia, MO. Two support groups in the USA publicized the study and referred interested members: Recurrent Respiratory Papillomatosis Foundation in Lawrenceville, NJ, and the International RRP Information, Support and Advocacy (ISA) Center in Bellingham, WA. The coordinating site was located at Allegheny General Hospital, Pittsburgh, PA. The study was approved at the coordinating center by the Institutional Review Board (IRB) named “Allegheny Singer Research Institute-WPAHS IRB” under research protocol number 4406. The research activities in Norfolk, Dallas and Cincinnati were overseen by the following IRBs respectively: Eastern Virginia Med School IRB, U of Texas Southwestern Med Ctr at Dallas IRB, Cincinnati Children's Hosp Med Ctr IRB. The activities of Dr. Lollar in Missouri were overseen by Allegheny Singer Research Institute-WPAHS IRB as agreed to in a registered section 114 of 45CFR46 cooperative research agreement with U of Missouri—U Hospitals and Clinics IRB. Dr. Bastian is in private practice and he was overseen by Allegheny Singer Research Institute-WPAHS IRB. All subjects provided written documentation of informed consent.

Eligible patients included those with histopathologic confirmation of a respiratory papilloma. Subjects were included regardless of degree of disease aggressiveness. Aggressiveness is a composite measure defined as any one of the following: ten or more total surgeries, four or more surgeries per year, distal spread beyond the subglottis, or ever requiring a tracheotomy [11]. Patients in remission for > 18 months could enroll, but the study capped the number of such patients to 10 due to finite resources. Excluded patients consisted of those with any of the following: splenectomy, immune disorders, immunosuppression, coagulopathy, or transfusion of immune globulin or blood in the past 6 months.

The researchers stratified the enrolled patients into four groups including: (1) patients receiving management for active disease who were > 11 years of age who never received a dose of the qHPV vaccine (adults/adolescents), (2) patients receiving management for active disease who were ≤ 11 years of age who never received a dose of the qHPV vaccine (children), (3) patients receiving management for active disease or in remission who were of any age and who had received at least one dose of the qHPV vaccine (qHPV vaccine), and (4) patients of any age in remission for > 18 months and who had never received a dose of the qHPV vaccine (remission). In summary, the first two groups were not in remission; they had active disease. The fourth group was in remission. The third group had had at least one dose of the qHPV vaccine and assignment to that group was independent of whether or not the patient had active disease or was in remission. Those patients who were vaccinated received the vaccine of their own free will prior to enrollment. These patients did not obtain the vaccine as an intervention of this study. Patients only had one visit during the study at which serum and clinical, as well as demographic, information were collected via a standard questionnaire. MSD Laboratories tested the serum samples for anti-HPV6 and 11 antibodies via their in-house competitive Luminex Immunoassay (cLIA)[12–14].

The protocol enrolled subjects regardless of if and when their next papilloma debridement would take place, and the protocol did not include collection of papillomas. We thus did not conduct HPV typing as part of this protocol and do not know if any one individual was infected by HPV6 or 11. Regardless, we reasonably expect that each subject had at least HPV6 or 11 and that a subject who was seronegative to both viruses (so called “double seronegative”) was almost certainly seronegative to the virus that had caused his or her papillomatosis. Most reported case series that included HPV typing revealed a very low frequency of HPV types other than 6 or 11. Patients were considered double seronegative if the geometric mean titre (GMT) in serum, for anti-HPV6 and anti-HPV11 antibodies was less than 20 milli Merck units per ml (mMu/ml) and 16 mMu/ml, respectively.

For 14 subjects, HPV type data was obtained from sources outside of this particular protocol. The typing data is reflected in the HPV6 vs HPV11 vs HPV611 (ie. Positive for both HPV6 and 11) facets of the figures. The outside data source was a parallel study named “Genetic Susceptibility to Papilloma-induced Voice Disturbance” and was also coordinated by Allegheny General Hospital. Subjects consented to the data from the genetic susceptibility study being used for this analysis.

Data was analyzed using R[15] and various packages: data.table[16], ggplot2[17], ggbeeswarm[18], BayesianFirstAid[19]. The dataset was initially inspected by conducting a descriptive statistical summary of all variables in isolation, inspecting boxplots and histograms of each variable. Variables that did not follow a Gaussian distribution were transformed by taking the natural logarithm of the value (serological titres, age at phlebotomy, duration of symptoms, duration from diagnosis to phlebotomy, time between last surgery for RRP and phlebotomy, age at enrollment). Initial analyses consisted of a Fisher exact test of independence for categorical variables. Multiple logistic regression was used to analyze multiple independent variables as a function of seropositivity. Where subjects were seropositive their titres were compared with t tests.

We used both Frequentist and Bayesian statistics. Frequentist statistics applies the commonly used null hypothesis significance testing utilizing p-values. In doing so, it essentially works backwards by calculating the probability of our collected data if the null hypothesis were to be true. Bayesian statistics, works forward by calculating the probability of the hypothesis given the data. Bayesian output is less susceptible to confident misunderstanding and misuse as is common with p-values[20]. The 95% high density interval was used as the 95% credible interval. No adjustments have been made for multiple testing.

## Results

A total of 70 patients were enrolled in the study. There were comparable numbers of males (56%) and females (44%), most of which were white (81%) (Table 1). The proportion of subjects whose clinical course had been aggressive was statistically similar among all groups (Fisher’s exact test for count data: p = 0.89) (Table 2).

**Table 1:** Demographic and clinical information of 70 patients with RRP.

	RRP Subjects (N = 70)
**Gender**	
Female	31 (44%)
Male	39 (56%)
**Race/Ethnicity**	
Asian/Pacific Islander	1 (1%)
Black/African American	3 (4%)
White	57 (81%)
Other/Multiracial	6 (9%)
Declined to respond	2 (3%)
Unknown	1 (1%)

**Table 2:** Demographic, clinical and titre information of four stratified groups. GMT is geometric mean titres measured in mMu/ml and is provided together with the interquartile range (IQR).

	adult+adlc	Child	qHPV vac	Remisn
	n = 36 Median (Range)	n = 16 Median (Range)	n = 8 Median (Range)	n = 10 Median (Range)
**Age at Phlebotomy (Years)**	44 (14–84)	4 (1–9)	32 (11–65)	38 (7–73)
**Age at Diagnosis (Years)**	31 (1–77)	2 (0–5)	15 (1–35)	25 (1–69)
**Duration of Symptoms (Years)**	11 (1–69)	3 (0–8)	9 (3–58)	9 (5–32)
**Time since last RRP surgery (Days)**	93 (14–768)	57 (20–359)	129 (4–1834)	1050 (599–8160)
**Clinical Course (Aggressive vs Indolent)**	61%	69%	75%	60%
**anti-HPV 6 titre** (when positive) GMT with IQR	88 (31–156) n = 16	137 (82–286) n = 3	400 (138–883) n = 8	51 (28–102) n = 3
**anti-HPV11 titre** (when positive) GMT with IQR	98 (43–193) n = 7	n = 0	446 (147–1588) n = 7	16 n = 1

**Fig 1:**
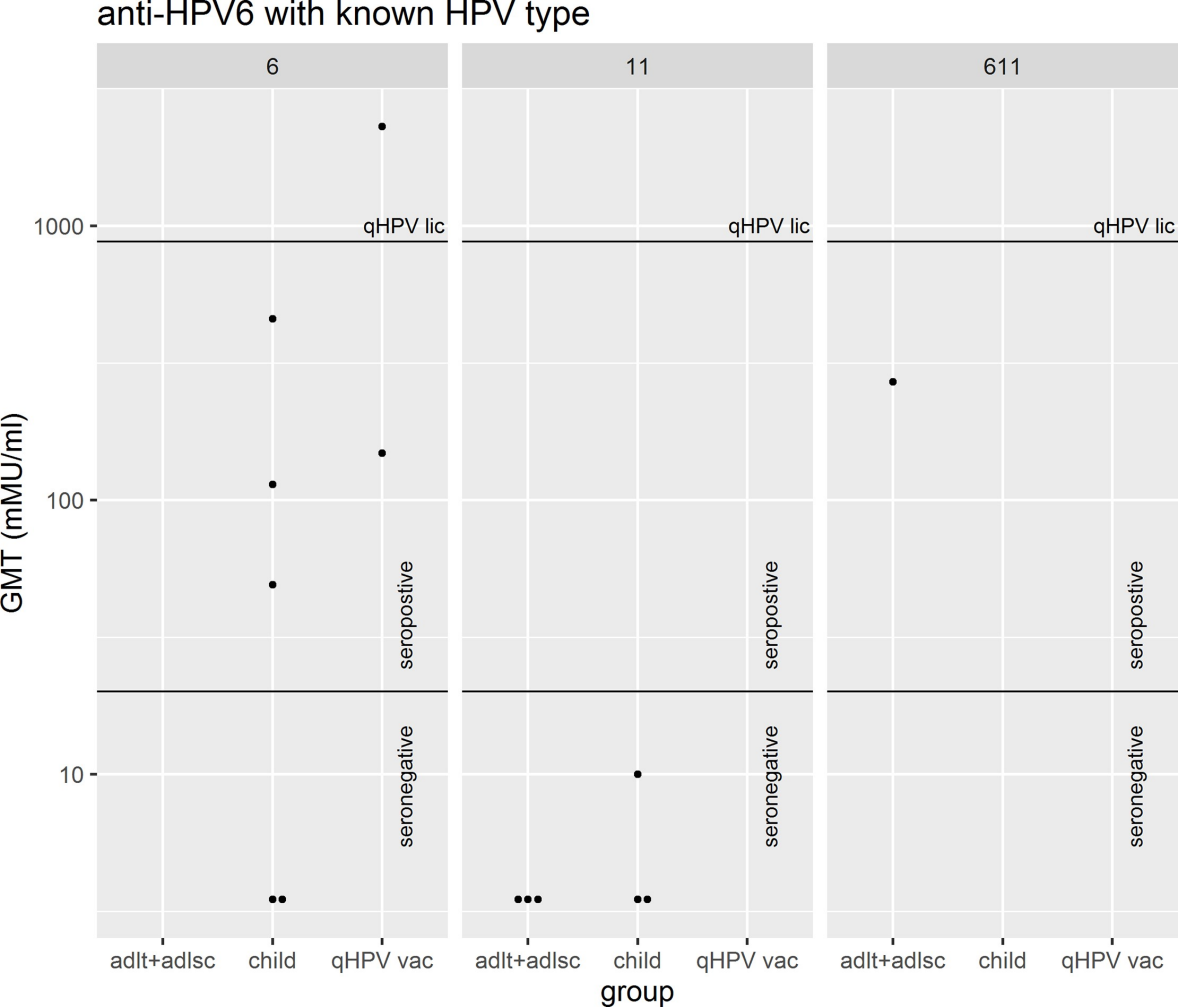
The GMT of anti-HPV6 antibodies for each individual of the specified subgroups faceted by HPV typing of papilloma where available. This figure illustrates the GMT for antibodies against HPV6 for all participants after stratification. A GMT above 20 milli Merck units (mMu) was considered positive. The line labeled “qHPV licens” represents the cLIA anti-HPV6 GMT at 1 month post third dose of qHPV vaccination among women 16–26 years of age in a clinical efficacy trial (Month 7 measurement in the trial) [21]. “adlt+adlsc” = Adult/Adolescent, “qHPV vac” = qHPV vaccine, “remisn” = remission. Column “6” indicates that the papilloma was infected with HPV6. Column “11” indicates that the papilloma was infected with HPV11. Column “611” indicates that the papilloma was coinfected with HPV6 and HPV11.

**Fig 2:**
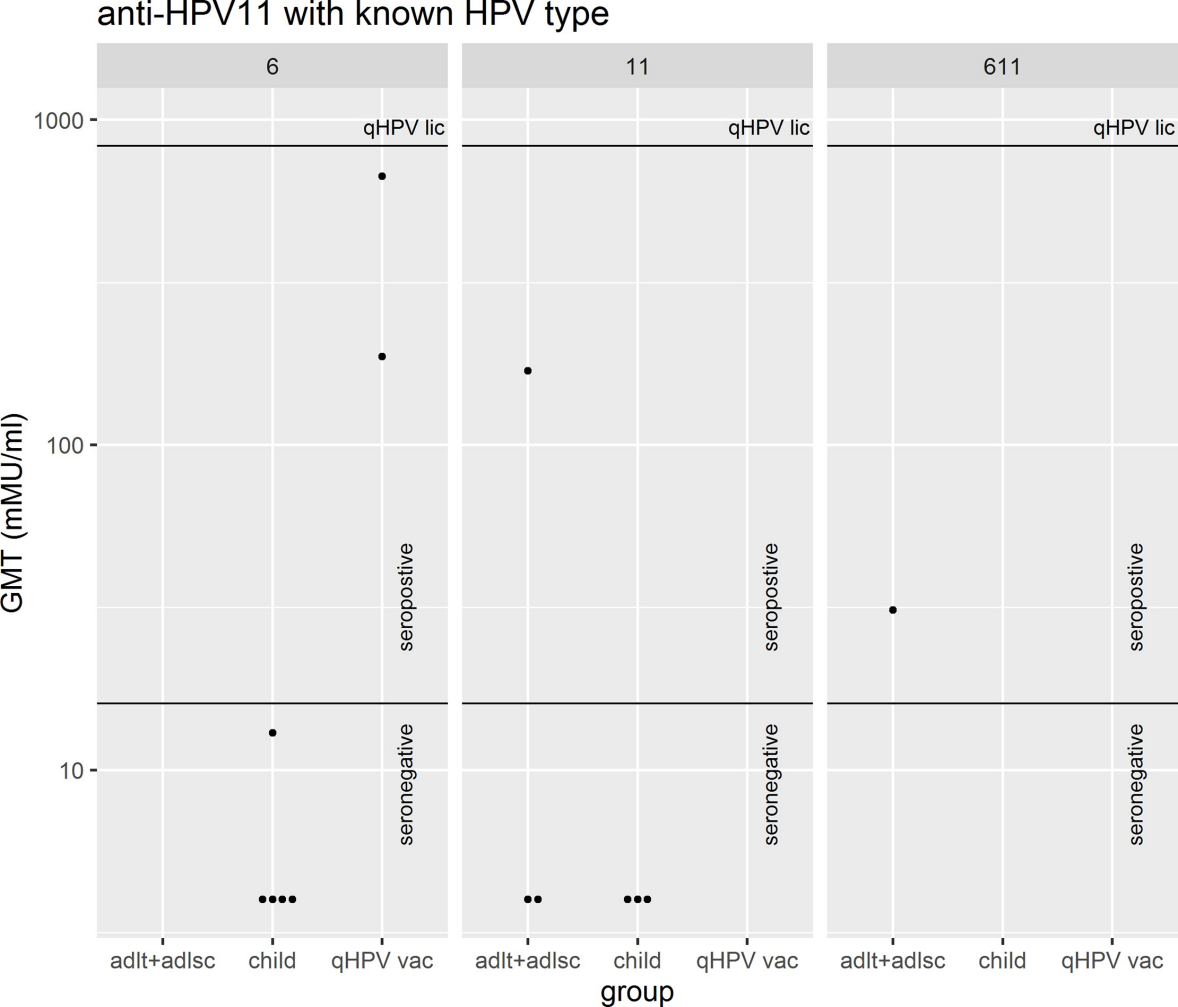
The GMT of anti-HPV11 antibodies for each individual of the specified subgroups faceted by HPV typing of papilloma where available. This figure illustrates the GMT for antibodies against HPV11 for all participants after stratification. A GMT above 16 milli Merck units (mMu) was considered positive. The line labeled “qHPV licens” represents the cLIA anti-HPV11 GMT at 1 month post third dose of qHPV vaccination among women 16–26 years of age in a clinical efficacy trial (Month 7 measurement in the trial) [21]. “adlt+adlsc” = Adult/Adolescent, “qHPV vac” = qHPV vaccine, “remisn” = remission. Column “6” indicates that the papilloma was infected with HPV6. Column “11” indicates that the papilloma was infected with HPV11. Column “611” indicates that the papilloma was coinfected with HPV6 and HPV11.

**Fig 3:**
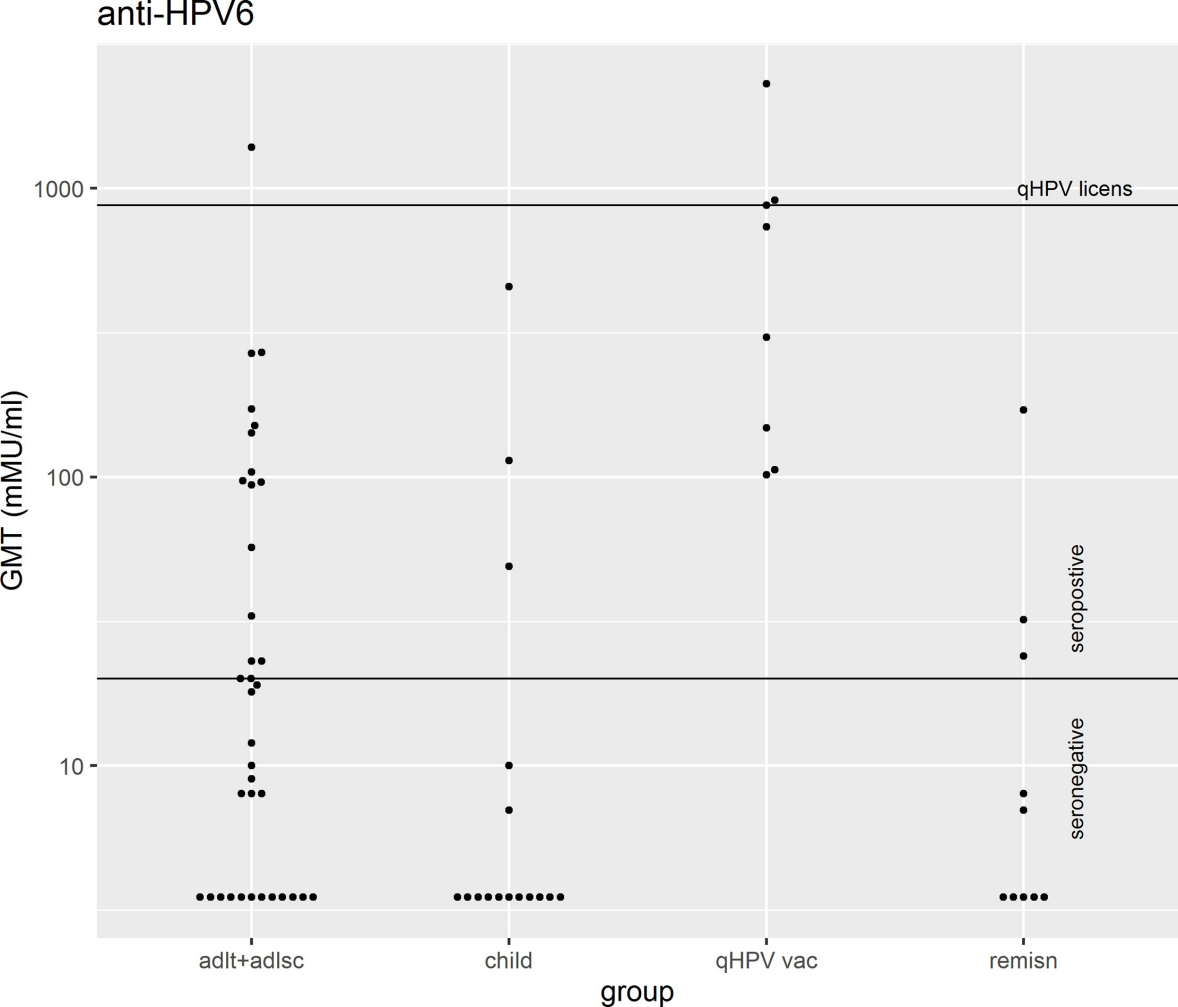
The GMT of anti-HPV6 antibodies. This figure illustrates the GMT for antibodies against HPV6 for all participants after stratification. A GMT above 20 milli Merck units (mMu) was considered positive. The line labeled “qHPV licens” represents the cLIA anti-HPV6 GMT at 1 month post third dose of qHPV vaccination among women 16–26 years of age in a clinical efficacy trial (Month 7 measurement in the trial) [21]. “adlt+adlsc” = Adult/Adolescent, “qHPV vac” = qHPV vaccine, “remisn” = remission.

**Fig 4:**
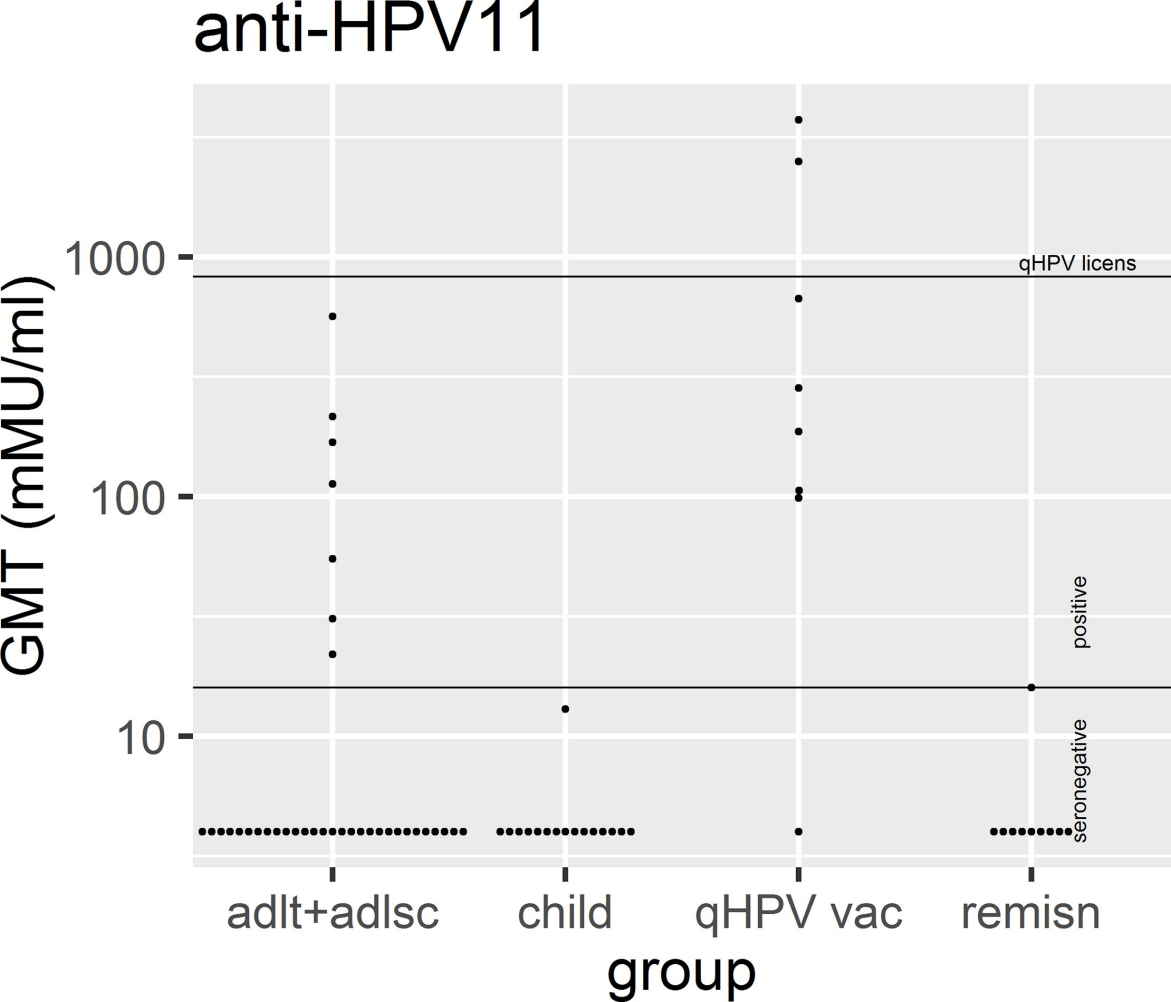
The GMT of anti-HPV11 antibodies. This figure illustrates the GMT for antibodies against HPV11 for all participants after stratification. A GMT above 16 milli Merck units (mMu) was considered positive.The line labeled “qHPV licens” represents the cLIA anti-HPV11 GMT at 1 month post third dose of qHPV vaccination among women 16–26 years of age in a clinical efficacy trial (Month 7 measurement in the trial) [21]. “adlt+adlsc” = Adult/Adolescent, “qHPV vac” = qHPV vaccine, “remisn” = remission.

The anti-HPV6 and anti-HPV11 titres are displayed in the figures. The first couple of figures are for a subset of subjects (n = 14) in which the HPV type was known (Fig 1, Fig 2). For 80% of all subjects the HPV type was not known; nevertheless, valuable information can be garnered by exploring double seronegative rates (see “Methods”). The next couple of figures are the titres for all 70 subjects (Fig 3, Fig 4).

**Fig 5:**
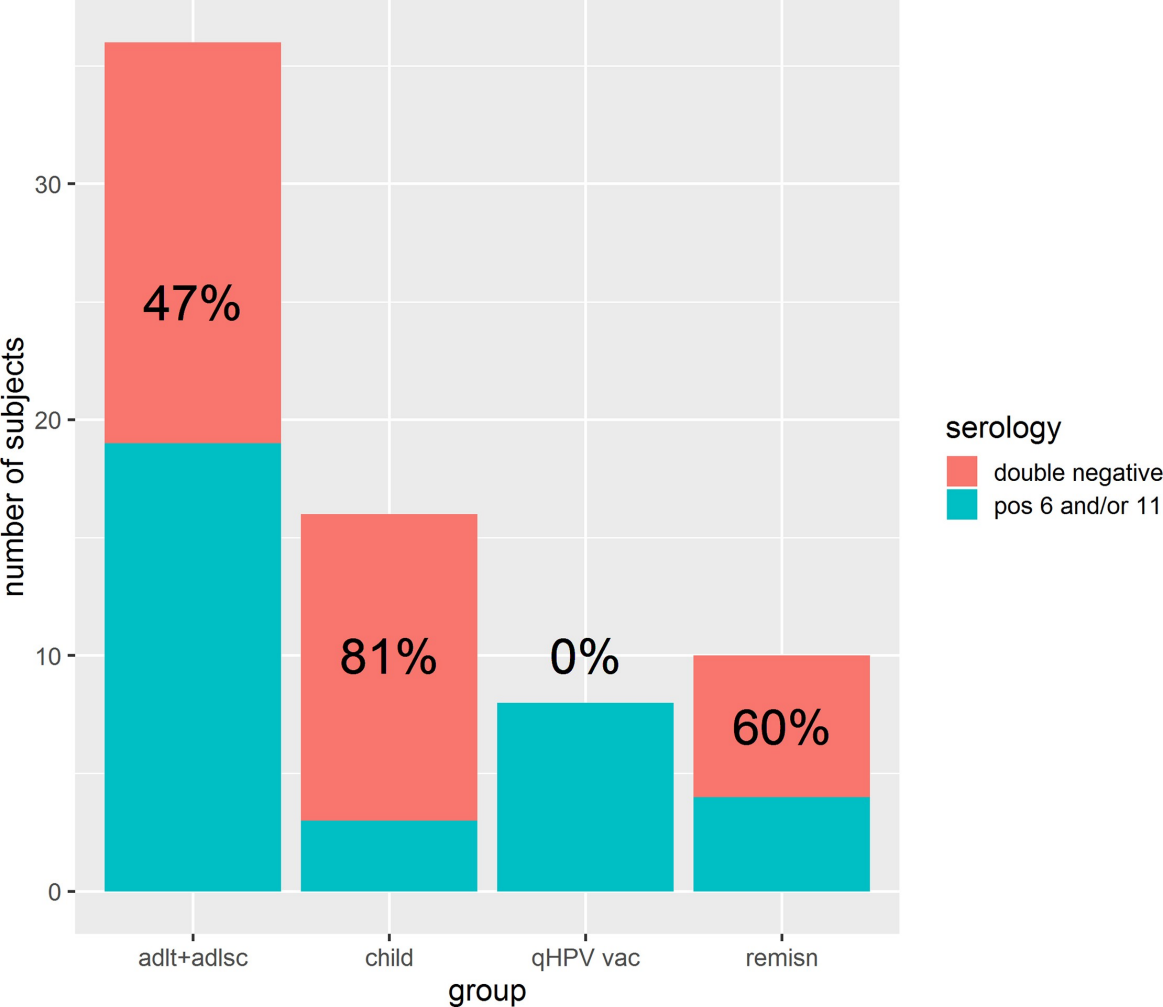
Serology data of stratified groups. This figure illustrates the serological responses of all 70 patients after being stratified into the four designated groups.

Eighty one percent (95% confidence interval [CI], 58% to 94%) of children and 47% (95% CI, 32% to 63%) of adults/adolescents were double seronegative (Fig 5). The difference in seronegativity was statistically significant between these two groups (odds ratio 0.2, Fisher’s exact test p = 0.03). The Bayesian estimate for the excess double seronegative among the children was 31% (95% credible interval 5.6% to 54%).

We had wondered if the nature of the clinical course (aggressive vs. indolent) was associated with serologic status. We compared GMTs and double seronegativity rates among those with different clinical courses; there was no statistically significant difference (data not presented).

**Fig 6:**
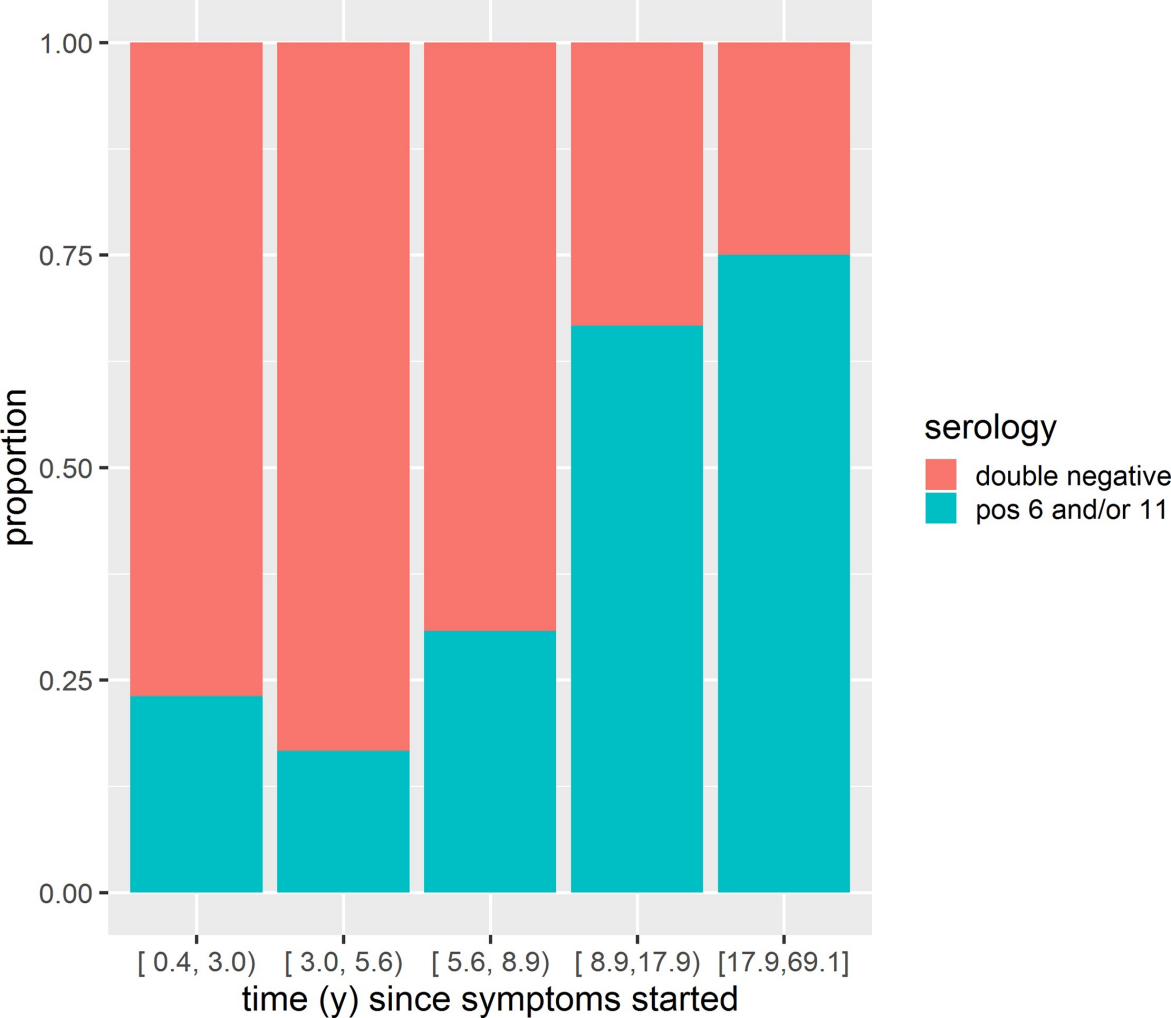
Time since symptoms started for patients who had never received the qHPV vaccine. This figure depicts the time since the patients’ symptoms started from when they received their phlebotomy for the study (n = 62). The patients were divided into equal quintile bins. The proportion of patients who were seropositive increased as the time since their symptoms began increased.

Multiple logistic regression models were used to explore associations with seropositivity for HPV6 and 11 among those who had not received the qHPV vaccine. There was a highly statistically significant association (p = 0.004) between the duration of symptoms (number of years between symptoms first occurring and the serology specimen being drawn) and the proportion who were seropositive (estimate of slope = 0.88, z value = 2.9) (Fig 6). Expressed differently, the geometric mean duration of symptoms in the 26 subjects who were seropositive was 11 years which is significantly longer (p = 0.0014) than the 4.7 years in the 36 subjects who were double seronegative. The strong association remained even when controlling for juvenile versus adult onset, and when controlling for age of the subject at the time of phlebotomy (modeled both dichotomously (≤11 years) and as a continuous variable). Much of the difference seen between the children and adults/adolescents groups was driven by the fact that the children had shorter duration of symptoms at the time of their enrollment. In a multiple logistic regression limited to only the two groups with active RRP and no use of the vaccination, only the duration of symptoms remained a statistically significant predictor of serologic status.

Among the 10 subjects in remission, 60% (95% CI, 31% to 83%) were double seronegative. For those who had never had the vaccine, the remission group were not demonstrably different to those with active disease with respect to serostatus. There was no discernable association between being double seronegative and the length of the remission (n = 10, logistic regression odds ratio 1.01 for each additional year since last surgery, p = 0.91). The median time since the last procedure for removal of papillomas among those that were double seronegative was 2.9 years and for seropositive it was 2.7 years (Wilcoxon rank sum test: p = 0.8). The geometric mean time since the last procedure was 3.6 years for the 6 people who were double seronegative as well as for the 4 people who were seropositive for HPV6 or HPV11 (Welch two sample t-test: p = 0.99). With Bayesian statistics, the estimate of the effect size was very small; there is only a 25% probability that the time since the last procedure is greater among those that are double seronegative with a medium effect size or larger (Cohen’s d ≥ 0.433).

None of the patients who were previously vaccinated with the qHPV vaccine (n = 8) were double seronegative (Fig 5). The Bayesian 95% credible interval was 0.004% to 28%. These 8 patients possessed antibodies to both HPV6 and 11, except one who was seronegative for HPV11. In free text comments, that particular subject volunteered information that he had received the first dose of the qHPV vaccine just 26 days before his blood draw. He was in his late 30s. We did not systematically elicit the details of those who had the qHPV vaccine; we know neither the number nor the date of administrations. Nevertheless, given that the vaccine became available in the USA in June 2006, the youngest anyone of the vaccinees could have been was 9.1 years old and the oldest possible age of administration would have been 65 years. Among the 8 subjects who had received qHPV vaccine, their anti-HPV6 GMT was 403 mMu/ml (Bayesian 95% credible interval 134 to 1212) which trended a little lower, geometrically speaking, than the 875 mMu/ml among healthy 16 to 26 year-old females at month 7 of the 3 doses in the pivotal qHPV vaccine trials (one sample Bayesian-t-test vs point estimate: p = 0.07) (Fig 3) [21]. The vaccinated group’s mean titre was also much greater than those 22 subjects with RRP who were seropositive against HPV6 but were never vaccinated, whose GMT was 81 mMu/ml (two-sample Bayesian t-test: p = 0.009, effect size = 1.2 which is very large). Among the 7 subjects who had undergone vaccination and were seropositive against HPV11; their GMT was 446 mMu/ml (95% CI 99 to 2208) and was only a little lower than the 830 mMu/ml that was the mean at month 7 of the 3 dose pivotal efficacy trials (one sample Bayesian t-test vs point estimate: 83% of the simulations resulted in the RRP vaccines being lower than the point estimate and 17% were greater than the point estimate (Fig 4). The mean titre among the vaccinated was higher than among the 8 people with RRP who were HPV11 seropositive (GMT was 74 mMu/ml (two-sample Bayesian t-test: p = 0.038, effect size = 1.1 which is very large)). Among the 8 vaccinees, 8 were HPV16 seropositive and 3 were HPV18 seropositive.

## Discussion

We have assembled a cross-sectional convenience sample covering a varied representation of stages within the clinical course of RRP. In so doing we have seen that many, if not most people with active RRP are seronegative to that which is causing their recurrent papillomas. However, the subjects with longer duration of symptoms had a higher proportion who were seropositive. We see that people with RRP are capable of the same robust response to the HPV vaccine as people who do not have RRP. Finally, seropositivity does not appear to be necessary to enter into remission; 60% of those in remission were seronegative (assuming that they did not lose their seropositivity over time).

The most significant finding of the study is that many patients with RRP were seronegative to a virus causing their disease. From our data, it is not clear if the “child” group were mostly seronegative because they were immunologically immature or if they simply had had less time to seroconvert. Multiple logistic regression would argue that perhaps the latter predominates; when controlling for both age at phlebotomy and duration since symptoms started, only duration of symptoms was significant. Children, particularly those with younger age at diagnosis tend to run a more aggressive course [22]. In our analysis, looking at all who had not received the vaccination, we saw an association of serologic status with neither aggressive clinical course nor age at the time of phlebotomy. Most people who develop RRP go into remission but it takes a long time to do so. It would be reasonable to believe that one needs an immunologic response to bring about remission and that such response would include a serologic manifestation. The data from the remission group is sparse, but to the extent that we have data, there is no evidence that seropositivity is necessary for remission to occur. More compelling data would necessitate that people with RRP undergo longitudinal immunologic assessments of both cellular and humoral immunity while they are in the active phase of the disease and be followed through remission.

We are aware of several earlier studies that investigated antibody responses in RRP. The results have been varied and that is not surprising given that there is no commercially available serologic test that could have served as a standard. Each laboratory designed and executed their own test and thus one can expect varying sensitivity and specificity. Bonnez et al. [23], working with predominantly children, reported that 15 out of 32 (47%) subjects were seropositive to an enzyme-linked immunosorbent assay (ELISA) with HPV11 virons. However, in their sample, 59% had HPV11 in the papillomas. Presumably most of those affected by HPV11 were seropositive which is at odds with our findings. Differences could be due to different reagent sensitivities and specificities or the differences of a cLIA versus an ELISA assay. The cLIA assay has gone through optimization and validation and used with many thousands of samples in licensing studies overseen by the FDA. Specifically it was optimized to "discriminate the low-titer antibody response of HPV-infected persons from noninfected individuals" [14].

Tachezy et al [24], working with predominantly adults, reported no difference in serostatus between cases and controls. However, they noted an association between the number of surgeries subjects had undergone and the optical density of their ELISA. We similarly found that the duration of symptoms was associated with seropositivity; duration of symptoms and number of surgeries are closely correlated. Tachezy et al’s findings encouraged us to vigorously interrogate our data. Duration and surgical count correlated with seronegative vs seropositive but not with titre given seropositivity. We were thus surprised when Durzyńska et al [25] reported a very tight correlation between ELISA absorbance and surgical count; an extraordinary 87% of the variance in absorbance could be predicted by the variance in surgical count. They only tested anti-HPV11 and 30% of their 47 subjects were not infected with HPV11.

Maloney et al [26], working with children, found, similar to us, that only 3 out of 15 were seropositive. However unlike in our study, all 3 of their positives were anti-HPV11 and none were anti-HPV6.

Some have wondered if people with RRP have a specific immunodeficiency to the HPV type that caused their RRP. If that were the case then it would be reasonable to expect that those with RRP may not mount a serologic response to the HPV vaccine as people without RRP do. Such expectations are not the case; the 8 people who had undergone vaccination were robustly seropositive in that their GMTs were closer to subjects in the vaccine trials than they were to those with active RRP. An absolute immunodeficiency thus is unlikely but we cannot exclude a relative humoral immunodeficiency (in strength and/or timing) or a cellular immunodeficiency. The single seronegative result was to HPV11 in a subject who had had their first dose 3½ weeks earlier; perhaps that is neither sufficient time nor sufficient number of doses to mount a robust immune response.

While the HPV vaccine efficacy in ano-genital HPV prevention is well established, its utility in treatment is viewed by many with skepticism. The cell biology of how neutralizing antibodies prevent infection[27] has been established but it does not include a pathway that would predict eradication of established infection, particularly high-risk HPV, and clinical studies in cervical infection[28,29] and genital warts[30] confirm as much. For several decades, Bonagura et al have investigated the cellular humoral response in RRP [31]. Their work suggests that the infection persists because of a HPV-specific downregulated innate and adaptive T cell response which is T_h_2/regulatory T cell weighted. The manifestations are seen both locally within the papilloma and systemically. Tolerance ensues rather than an inflammatory response that would clear the infection by killing affected keratinocytes.

Notwithstanding the prevailing biological understanding of viral clearance, the potent humoral response to the vaccine in contrast to the absent or weak response to natural infection has attracted interest. As could be expected, the vaccine has aroused hope in those desperately seeking an intervention and who are less encumbered by a profound understanding of cellular immunology mechanisms. Several investigators have indeed pursued the possibility of using an HPV vaccine for treatment. Compared to historical controls, Zhang witnessed regression of genital warts in response to HPV6 VLP administration[32]. In dogs, the canine oral papilloma virus reliably causes papilloma on the oral mucosal surface and in almost all dogs the lesions spontaneously regress around 6 weeks[33]. Rarely, the papillomas persist and become chronically exuberant. There have been reports of such animals resolving their disease after administration of VLP made of the L1 protein of COPV[34,35].

Given the safety of the HPV vaccine, multiple doctors and patients have attempted to, empirically and in an off-label manner, administer the vaccine in people with RRP. Some surgeons have conducted such interventions under a research protocol and have published their observations. Several showed a serologic response [36,37] to the vaccine and an increase in the time between surgeries [5,6,38,39]. Hermann et al [7] and Milner et al [8]found no such difference. All of these studies are limited by their small sample sizes and the absence of randomized controls. RRP is a disease with a variable natural history and even without a non-surgical intervention (often referred to in the RRP literature as “adjuvant” therapy) most people trend towards less frequent surgery [40] and then remission. This makes RRP case series without controls and without blinding particularly beguiling.

As previously alluded to, researchers have postulated about why some patients are unable to successfully fight off infection with HPV. More research must be done to elucidate the cause for this very specific immunodeficiency in patients with RRP; however, insight can be gained by looking at well-established research on HPV infection in the genital tract and parallels can be drawn to HPV infection in the respiratory tract. Similar to RRP, genital HPV6 and 11 disease is an indolent infection of the epithelium. According to Stanley, immune responses to HPV6 and 11 infections are weak and slow to develop in the genital tract, with a mean duration of infection between 8 and 9 months [41]. Our results lend support to this understanding, as the proportion of patients who were seropositive increased as the time since symptom onset increased.

Unlike most other research studies regarding serological RRP data, our study is unique in that it includes: a more expansive sample size, both male and female patients, and data in terms of the onset of disease rather than just current age, includes people with RRP that are in remission, and includes comparisons to point estimates of antibody titres from a cohort of people without RRP.

Our study has limitations. This was simply a cross-sectional observational study of a convenience cohort without interventions. Our cohort is probably enriched for aggressive cases since such subjects are more likely to be invited by their treating physician and/or more likely to volunteer. Most of the clinical data was elicited from patients or their parents and thus subject to recall bias. For those who received the qHPV vaccine of their own free will, we do not know the circumstances of their administrations (e.g., number of doses or age at which it was received) and yet, conservatively, we compare them to subjects aged 16 to 26 years in pivotal efficacy trials where complete and timely administration was ensured and where serology was determined uniformly at 7 months since the first dose [21]. In the pivotal efficacy trials, at 12 months since the first dose, antibody levels against HPV 6 and 11 were 319 mMU/mL and 264 mMU/mL, respectively [21].

Future research should focus on filling the gaps that exist: longitudinal studies, cellular immunity and control groups. With regards to the potential of the vaccine to cure RRP or diminish the burden, there must be a blinded randomized controlled trial. While serology addresses the humoral arm of the immune response, it is widely believed that the killing of mucosal cells bearing HPV episomes for cure would require the cellular arm. Future studies would do well to include measures of such response. Finally, almost nothing is known about the physiology that permits those with RRP to go into remission. Longitudinal studies that follow subjects through active disease and into remission would probably illuminate the process and potentially enable us to precipitate such pathway prematurely.

Over the last decade, many countries have included qHPV or nine-valent vaccination in their routine immunization schedule. The primary motivation and justification was the reduction in HPV-related anogenital disease and resulting deaths. RRP is rare and has no suitable clinical proxy precursor (unlike cervical intraepithelial neoplasia) and thus, to study its prevention in a clinical trial is challenging. Nevertheless, several initiatives are exploring the incidence of RRP as a function of qHPV vaccination at the population level. Data from Australia has shown a reduction in the incidence of juvenile-onset RRP after implementation of the National HPV Vaccination Program[42]. Such data is compatible with, and therefore supportive of, our previously-held reasonable expectation that vaccination has the potential to prevent this devastating disease.
